# Antinociceptive properties of new coumarin derivatives bearing substituted 3,4-dihydro-2*H*-benzothiazines

**DOI:** 10.1186/2008-2231-22-9

**Published:** 2014-01-07

**Authors:** Masoumeh Alipour, Mehdi Khoobi, Saeed Emami, Saeed Fallah-Benakohal, Seyedeh Farnaz Ghasemi-Niri, Mohammad Abdollahi, Alireza Foroumadi, Abbas Shafiee

**Affiliations:** 1 School of Chemistry, University College of Science, University of Tehran, P.O. Box 14155–6455, Tehran, Iran; 2 Department of Medicinal Chemistry, Faculty of Pharmacy and Pharmaceutical Sciences Research Center, Tehran University of Medical Sciences, Tehran 14176, Iran; 3 Department of Medicinal Chemistry and Pharmaceutical Sciences Research Center, Faculty of Pharmacy, Mazandaran University of Medical Sciences, Sari, Iran; 4 Department of Toxicology and Pharmacology, Pharmaceutical Sciences Research Center, Faculty of Pharmacy, Tehran University of Medical Sciences, Tehran 14176, Iran

**Keywords:** Analgesic activity, Antinociception, Coumarin, Benzothiazine, Formalin test, Writhing test

## Abstract

**Background:**

Coumarins are an important class of widely distributed heterocyclic natural products exhibiting a broad pharmacological profile. In this work, a new series of coumarins bearing substituted 3,4-dihydro-2*H*-benzothiazines were described as potential analgesic agents. The clinical use of NSAIDs as traditional analgesics is associated with side effects such as gastrointestinal lesions and nephrotoxicity. Therefore, the discovery of new safer drugs represents a challenging goal for such a research area.

**Results:**

The target compounds 3-(3-methyl-3,4-dihydro-2*H*-benzo[*b*][1,4]thiazin-3-yl)-2*H*-chromen-2-ones **2a-u** were synthesized and characterized by spectral data. The antinociceptive properties of target compounds were determined by formalin-induced test and acetic acid-induced writhing test in mice. Among the tested compounds, compound **2u** bearing 2-(4-(methylsulfonyl)benzoyl)- moiety on benzothiazine ring and 4-(methylsulfonyl)phenacyloxy- group on the 7 position of coumarin nucleus showed better profile of antinocecieption in both models. It was more effective than mefenamic acid during the late phase of formalin-induced test as well as in the acetic acid-induced writhing test.

**Conclusion:**

Considering the significant antinoceciptive action of phenacyloxycoumarin derivatives, compound **2u** prototype might be further used as model to obtain new more potent analgesic drugs.

## Introduction

Pain is an uncomfortable sensation that alerts the human organs about a current or potential damage to tissues [1]. It has been accepted that pain can widely affect human life quality, and its management is considered as a main challenge in pharmacotherapy [2]. NSAIDs are one of major classes of traditional analgesics for treatment of pain. The clinical use of NSAIDs is associated with side effects such as gastrointestinal lesions and nephrotoxicity [3]. Therefore, the discovery of new safer drugs represents a challenging goal for such a research area.

Coumarins are an important class of widely distributed heterocyclic natural products exhibiting a broad pharmacological profile [4]. Several coumarin derivatives have been synthesized with diverse biological activities [5-9] especially analgesic/anti-inflammatory activity [10-13]. Recently, the synthesis and anti-inflammatory/analgesic activities of several coumarin derivatives with various substitutions on 3-position of coumarin nucleus have been reported [14-16]. On the other hand, benzothiazine derivatives are also important heterocyclic compounds with wide spectrum of biological activities [17,18]. In view of the above facts and in continuation of our research program on the synthesis of biologically active heterocyclic compounds [19,20], we introduced herein the new coumarin derivatives bearing substituted 3,4-dihydro-2*H*-benzothiazines as analgesic agents. The antinociceptive properties of target compounds were determined by formalin-induced paw licking test and acetic acid-induced writhing test in mice. Indeed, the formalin-induced paw licking method is used to investigate both peripheral and central mechanisms whereas the acetic acid test is believed to demonstrate the involvement of peripheral mechanisms in the control of pain [21,22].

## Materials and methods

### Chemistry

**Scheme 1:**
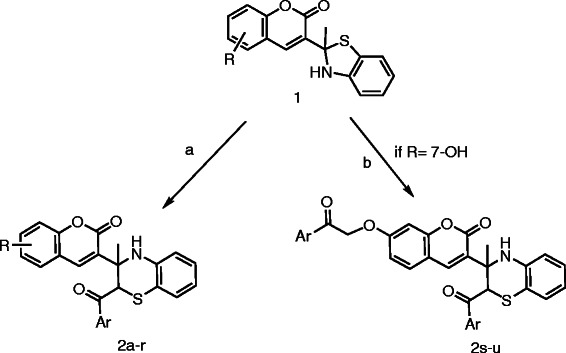
**Synthesis of coumarin based dihydrobenzothiazines 2a-u.** Reagents and conditions: (a) phenacyl halide (1.2 mmol), KF/Al_2_O_3_ (0.7 g), quinine hydrochloride (10 mol%), EtOH (3 mL), r.t. (b) phenacyl halide (2.5 mmol), KF/Al_2_O_3_ (1.5 g), quinine hydrochloride (10 mol%), EtOH (3 mL), r.t.

The target compounds 3-(3-methyl-3,4-dihydro-2*H*-benzo[*b*][1,4]thiazin-3-yl)-2*H*-chromen-2-ones **2a-r** (Additional file 1: Table S1) were synthesized according to the pathway outlined in Scheme 1[23]. All reagents and chemicals were commercially available and used as received. Alumina-supported potassium fluoride (KF/Al_2_O_3_) was prepared by literature method [24]. The dihydrobenzothiazole derivatives **1** were prepared as reported method by us [19,20]. The synthesis of compounds **2a-d**, **2f-i** and **2p-r** was described in our previous paper [23]. Column chromatography was carried out on silica gel (70–230 mesh). TLC was conducted on silica gel 250 micron, F254 plates. Melting points were measured on a Kofler hot stage apparatus and are uncorrected. The IR spectra were taken using Nicolet FT-IR Magna 550 spectrographs (KBr disks). ^1^H NMR spectra were recorded on a Bruker 400 or 500 MHz NMR instruments. The chemical shifts (δ) and coupling constants (*J*) are expressed in parts per million and Hertz, respectively. Mass spectra of the products were obtained with an HP (Agilent technologies) 5937 Mass Selective Detector. Elemental analyses were carried out by a CHN-Rapid Heraeus elemental analyzer. The results of elemental analyses (C, H, N) were within ± 0*.*4% of the calculated values.

### General procedure for the synthesis of compounds 2

A suspension of dihydrobenzothiazole derivatives **1** (1.0 mmol), KF/Al_2_O_3_ (0.7 g), and quinine hydrochloride (10 mol%) in ethanol (3.0 mL) was stirred at room temperature for 5 min. Then, appropriate phenacyl halide (1.2 mmol) was added to the mixture and stirring was continued. After completion of the reaction (3–5 h), the solvent was removed under reduced pressure. The residue was mixed with ethyl acetate (5 mL) and the catalyst was filtered and washed with ethyl acetate (3 × 5 mL). After evaporation of the solvent at reduced pressure, the crude product was purified by column chromatography (*n*-hexane/ethyl acetate, 9:1) and crystallized from ethanol for further purification.

#### 3-(2-(3,4-Dichlorobenzoyl)-3-methyl-3,4-dihydro-2H-benzo[b] [1,4]thiazin-3-yl)-2H-chromen-2-one (2e)

Yellow solid (361 mg, 75%); *syn*-isomer; mp 91–93°C; IR (KBr, cm^-1^) 3382 (NH), 1708 (C=O); ^1^H NMR (500 MHz, CDCl_3_) δ ^1^H NMR (500 MHz, CDCl_3_) δ 1.94 (s, 3H, CH_3_ benzothiazine), 4.49 (s, 1H, NH), 5.77 (s, 1H, C-H benzothiazine), 6.75 (dt, *J* = 7.2 and 1.2 Hz, 1H, H_7_ benzothiazine), 6.95 (m, 2H, H_5,8_ benzothiazine), 7.14 (dt, *J* = 7.2 and 1.2 Hz, 1H, H_6_ benzothiazine), 7.22 (t, *J* = 8.0 Hz, 1H, H_6_ chromene), 7.28 (dd, *J* = 8.0 and 1.9 Hz, 1H, H_5_ benzoyl), 7.33 (d, *J* = 8.0 Hz, 1H, H_6_ benzoyl), 7.40 (m, 2H, H_5,8_ chromene), 7.43 (d, *J* = 1.9, 1H, H_3_ benzoyl), 7.49 (dt, *J* = 8.0 and 1.2 Hz, 1H, H_7_ chromene), 7.77 (s, 1H, H_4_ chromene); ^13^C NMR (125 MHz, CDCl_3_) δ 24.3, 42.9, 58.0, 110.9, 116.1, 117.1, 119.1, 119.3, 124.3, 127.0, 127.1, 128.4, 128.7, 130.1, 130.8, 131.2, 131.4, 132.0, 136.9, 137.1, 139.8, 141.0, 153.2, 160.9, 192.6; Anal. calcd for C_25_H_17_Cl_2_NO_3_S: C, 62.25; H, 3.55; N, 2.90. Found: C, 62.41; H, 3.67; N, 3.15.

#### 3-(2-(4-Fluorobenzoyl)-3-methyl-3,4-dihydro-2H-benzo[b] [1,4]thiazin-3-yl)-2H-chromen-2-one (2j)

Yellow solid (336 mg, 78%); *syn*-isomer; mp 161–163°C; IR (KBr, cm^-1^) 3413 (NH), 1708 (C=O); ^1^H NMR (500 MHz, CDCl_3_) δ 1.76 (s, 3H, CH_3_ benzothiazine), 4.50 (s, 1H, NH), 5.97 (s, 1H, C-H benzothiazine), 6.73 (t, *J* = 7.4 Hz, 1H, H_7_ benzothiazine), 6.93 (m, 2H, H_5,6_ benzothiazine), 7.14 (m, 3H, H_8_ benzothiazine and H_3,5_ benzoyl), 7.28 (t, *J* = 7.4 Hz, 1H, H_6_ chromene), 7.35 (d, *J* = 7.4 Hz, 1H, H_8_ chromene), 7.40 (d, *J* = 7.4 Hz, 1H, H_5_ chromene), 7.50 (t, *J* = 7.4 Hz, 1H, H_7_ chromene), 7.79 (s, 1H, H_4_ chromene), 8.05 (m, 2H, H_2,6_ benzoyl); ^13^C NMR (125 MHz, CDCl_3_) δ 24.6, 37.5, 57.6, 111.9, 115.7, 115.9, 116.1, 119.1, 119.4, 124.4, 126.7, 128.4, 128.5, 131.2, 131.3, 131.5, 133.2, 139.5, 141.1, 153.3, 161.3, 164.7, 166.7, 191.3; Anal. calcd for C_25_H_18_FNO_3_S: C, 69.59; H, 4.20; N, 3.25. Found: C, 69.42; H, 4.03; N, 3.47.

#### 3-(3-Methyl-2-(thiophene-2-carbonyl)-3,4-dihydro-2H-benzo[b][1,4]thiazin-3-yl)-2H-chromen-2-one (2k)

Yellow solid (356 mg, 85%); as mixture of diastereomers (*anti/syn: *15*/*85); IR (KBr, cm^-1^) 3389 (NH), 1707 (C=O); ^1^H NMR (500 MHz, CDCl_3_) δ 1.77_syn_ (s, CH_3_ benzothiazine), 1.87_anti_ (s, CH_3_ benzothiazine), 4.48_syn_ (s, NH), 4.53_anti_ (s, NH), 5.50_anti_ (s, C-H benzothiazine), 5.87_syn_ (s, C-H benzothiazine), 6.77_syn_ (t, *J* = 8.0, H_7_ benzothiazine), 6.81_anti_ (t, *J* = 8.0, H_7_ benzothiazine), 6.92_anti_ (d, *J* = 8.0 Hz, H_5_ benzothiazine), 6.95_syn_ (d, *J* = 8.0 Hz, H_5_ benzothiazine), 7.07-7.10_anti_ (m, H_6,8_ benzothiazine), 7.10-7.13_syn_ (m, H_6,8_ benzothiazine), 7.20_syn_ (t, *J* = 7.6 Hz, H_7_ chromene), 7.25_syn_ (t, *J* = 7.6 Hz, H_6_ chromene), 7.25-7.29_anti_ (m, H_4_ thiophene and H_7_ chromene), 7.38_syn_ (d, *J* = 7.6 Hz, H_5_ chromene), 7.42_syn_ (d, *J* = 7.6 Hz, H_8_ chromene), 7.48-7.54 (m, H_5,7,8_ chromene (anti) and H_4_ thiophene (syn)), 7.56_anti_ (d, *J* = 4.0, H_3_ thiophene), 7.74_syn_ (d, *J* = 4.0, H_3_ thiophene), 7.79_anti_ (d, *J* = 4.0, H_5_ thiophene), 7.80_syn_ (s, H_4_ chromene), 7.97_syn_ (d, *J* = 4.0, H_5_ thiophene), 8.15_anti_ (s, H_4_ chromene); ^13^C NMR (*syn*-isomer, 125 MHz, CDCl_3_) δ 27.5, 45.3, 54.2, 116.1, 117.0, 118.6, 119.0, 120.1, 124.5, 126.2, 127.1, 128.2, 128.4, 131.4, 131.7, 132.2, 134.4, 140.2, 140.9, 143.5, 153.1, 160.1, 186.8; Anal. calcd for C_23_H_17_NO_3_S_2_: C, 65.85; H, 4.08; N, 3.34. Found: C, 65.92; H, 3.91; N, 3.29.

#### 3-(2-(5-Bromothiophene-2-carbonyl)-3-methyl-3,4-dihydro-2H-benzo[b][1,4]thiazin-3-yl)-2H-chromen-2-one (2l)

Yellow solid (378 mg, 77%); as mixture of diastereomers (*anti/syn: *26*/*74); IR (KBr, cm^-1^) 3390 (NH), 1712 (C=O); ^1^H NMR (500 MHz, CDCl_3_) δ 1.75_syn_ (s, CH_3_ benzothiazine), 1.87_anti_ (s, CH_3_ benzothiazine), 4.45_syn_ (s, NH), 4.55_anti_ (s, NH), 5.48_anti_ (s, C-H benzothiazine), 5.78_syn_ (s, C-H benzothiazine), 6.76_syn_ (t, *J* = 8.0, H_7_ benzothiazine), 6.81_anti_ (t, *J* = 8.0, H_7_ benzothiazine), 6.92_syn_ (d, *J* = 8.0 Hz, H_5_ benzothiazine), 6.94_anti_ (d, *J* = 8.0 Hz, H_5_ benzothiazine), 6.92_anti_ (d, *J* = 8.0 Hz, H_8_ benzothiazine), 6.96_syn_ (d, *J* = 8.0 Hz, H_8_ benzothiazine), 7.09_anti_ (t, *J* = 8.0 Hz, H_6_ benzothiazine), 7.12_syn_ (t, *J* = 8.0 Hz, H_6_ benzothiazine), 7.16_syn_ (d, *J* = 4.0 Hz, H_4_ thiophene), 7.23_anti_ (d, *J* = 4.0 Hz, H_4_ thiophene), 7.24_syn_ (t, *J* = 7.5, H_6_ chromene), 7.27-7.29_anti_ (m, H_6,8_ chromene), 7.36_syn_ (d, *J* = 7.5 Hz, H_8_ chromene), 7.40_syn_ (d, *J* = 7.5 Hz, H_5_ chromene), 7.50 _syn_ (t, *J* = 7.5 Hz, H_7_ chromene), 7.51-7.53_anti_ (m, H_5,7_ chromene), 7.56_anti_ (d, *J* = 4.0 Hz, H_3_ thiophene), 7.69_syn_ (d, *J* = 4.0 Hz, H_3_ thiophene), 7.77_syn_ (s, H_4_ chromene), 8.15_anti_ (s, H_4_ chromene); ^13^C NMR (*syn*-isomer, 125 MHz, CDCl_3_) δ 24.8, 38.7, 57.4, 112.3, 116.1, 116.9, 119.1, 119.5, 123.6, 124.4, 126.6, 128.1, 128.4, 130.8, 131.5, 131.6, 132.4, 139.2, 141.0, 145.9, 153.3, 161.2, 185.6; MS, *m/z* (%) 499 ([M + 2]^+^, 40%), 497 (M^+^, 37), 375 (47), 373 (44), 308 (100), 294 (51), 280 (84); Anal. calcd for C_23_H_16_BrNO_3_S_2_: C, 55.43; H, 3.24; N, 2.81 Found: C, 55.22; H, 3.47; N, 2.73.

#### 3-(3-Methyl-2-(thiophene-3-carbonyl)-3,4-dihydro-2H-benzo[b][1,4]thiazin-3-yl)-2H-chromen-2-one (2m)

Yellow solid (335 mg, 80%); as mixture of diastereomers (*anti/syn: *32*/*68); IR (KBr, cm^-1^) 3374 (NH), 1708 (C=O); ^1^H NMR (500 MHz, CDCl_3_) δ 1.77_syn_ (s, CH_3_ benzothiazine), 1.87_anti_ (s, CH_3_ benzothiazine), 4.50_anti_ (s, NH), 4.55_syn_ (s, NH), 5.48_anti_ (s, C-H benzothiazine), 5.81_syn_ (s, C-H benzothiazine), 6.74_syn_ (t, *J* = 7.5, H_7_ benzothiazine), 6.80_anti_ (t, *J* = 7.5, H_7_ benzothiazine), 6.93_anti_ (d, *J* = 7.5 Hz, H_5_ benzothiazine), 6.97_syn_ (d, *J* = 7.5 Hz, H_5_ benzothiazine), 7.08_anti_ (d, *J* = 7.5 Hz, H_8_ benzothiazine), 7.12_syn_ (t, *J* = 7.5 Hz, H_6_ benzothiazine), 7.13_syn_ (t, *J* = 7.2 Hz, H_6_ chromene), 7.24-7.25_syn_ (m, H_8_ benzothiazine and H_8_ chromene), 7.26-7.29_anti_ (m, H_6_ benzothiazine and H_6,8_ chromene), 7.35-7.37_syn_ (m, H_4_ thiophene and H_5,7_ chromene), 7.37-7.40_anti_ (m, H_6_ chromene and H_4_ thiophene), 7.50-7.53_anti_ (m, H_7_ chromene and H_5_ thiophene), 7.58_syn_ (d, *J* = 5.0 Hz, H_5_ thiophene), 7.79_anti_ (s, H_2_ thiophene), 8.04_syn_ (s, H_2_ thiophene), 8.14_anti_ (s, H_4_ chromene), 8.25_syn_ (s, H_4_ chromene); ^13^C NMR (*syn*-isomer, 125 MHz, CDCl_3_) δ 27.8, 45.7, 54.4, 116.1, 116.8, 119.1, 119.9, 124.6, 126.3, 126.5, 127.3, 128.2, 128.4, 130.6, 131.5, 131.7, 132.6, 140.0, 140.2, 141.2, 153.1, 160.2, 187.7; MS, *m/z* (%) 419 (M^+^, 68%), 404 (12), 386 (12), 308 (97), 295 (100), 280 (64), 111 (63); Anal. calcd for C_23_H_17_NO_3_S_2_: C, 65.85; H, 4.08; N, 3.34. Found: C, 65.98; H, 3.82; N, 3.60.

#### 8-Methoxy-3-(3-methyl-2-(4-methylbenzoyl)-3,4-dihydro-2H-benzo[b][1,4]thiazin-3-yl)-2H-chromen-2-one (2n)

Yellow solid (343 mg, 75%); *syn*-isomer; mp 145–147°C; IR (KBr, cm^-1^) 3360 (NH), 1700 (C=O); ^1^H NMR (500 MHz, CDCl_3_) δ 1.77 (s, 3H, CH_3_ benzothiazine), 2.43 (s, 3H, CH_3_ benzoyl), 3.90 (s, 3H, O-CH_3_ chromene), 4.52 (s, 1H, NH), 6.00 (s, 1H, C-H benzothiazine), 6.78 (dt, *J* = 7.5 and 1.3 Hz, 1H, H_7_ benzothiazine), 6.93 (dd, *J* = 8.0 and 1.3 Hz, 1H, H_7_ chromene), 7.02-7.11 (m, 4H, H_5,6,8_ benzothiazine and H_6_ chromene), 7.18 (m, 3H, H_5_ chromene and H_3,5_ benzoyl), 7.74 (d, *J* = 8.3 Hz, 2H, H_2,6_ benzoyl), 8.13 (s, 1H, H_4_ chromene); ^13^C NMR (125 MHz, CDCl_3_) δ 21.6, 27.9, 43.6, 54.6, 56.1, 113.3, 114.8, 118.9, 119.4, 119.7, 119.9, 124.3, 126.3, 127.4, 128.5, 129.3, 131.1, 133.7, 140.0, 140.2, 142.8, 143.8, 146.7, 159.5, 192.9 cm^-1^; Anal. calcd for C_27_H_23_NO_4_S: C, 70.88; H, 5.07; N, 3.06. Found: C, 70.64; H, 5.23; N, 3.22.

#### 3-(2-(4-Fluorobenzoyl)-3-methyl-3,4-dihydro-2H-benzo[b][1,4]thiazin-3-yl)-8-methoxy-2H-chromen-2-one (2o)

Yellow solid (323 mg, 70%); *syn*-isomer; mp 236–238°C; IR (KBr, cm^-1^) 3398 (NH), 1690 (C=O); ^1^H NMR (500 MHz, CDCl_3_) δ 1.78 (s, 3H, CH_3_ benzothiazine), 3.98 (s, 3H, O-CH_3_ chromene), 4.51 (s, 1H, NH), 5.98 (s, 1H, C-H benzothiazine), 6.74 (t, *J* = 7.4 Hz, 1H, H_7_ benzothiazine), 6.93 (m, 2H, H_5,6_ benzothiazine), 6.98 (d, *J* = 8.0 Hz, 1H, H_7_ chromene), 7.05 (m, 2H, H_5,6_ chromene), 7.16 (m, 3H, H_8_ benzothiazine and H_3,5_ benzoyl), 7.77 (s, 1H, H_4_ chromene), 8.05 (m, 2H, H_2,6_ benzoyl); ^13^C NMR (125 MHz, CDCl_3_) δ 24.5, 37.4, 56.3, 57.6, 111.9, 113.3, 115.7, 115.9, 116.9, 119.3, 119.8, 124.2, 126.7, 128.4, 131.2, 131.3, 131.4, 133.1, 139.5, 141.2, 146.8, 160.7, 164.7, 166.7, 191.2; Anal. calcd for C_26_H_20_FNO_4_S: C, 67.67; H, 4.37; N, 3.04. Found: C, 67.43; H, 4.18; N, 3.25.

#### 3-(2-(4-Bromobenzoyl)-3-methyl-3,4-dihydro-2H-benzo[b][1,4]thiazin-3-yl)-7-(2-(4-bromophenyl)-2-oxoethoxy)-2H-chromen-2-one (2s)

Yellow solid (507 mg, 72%); as mixture of diasteromers (*anti*/*syn*: 18/82); IR (KBr, cm^-1^) 3382 (NH), 1697 (C=O); ^1^H NMR (500 MHz, CDCl_3_) δ 1.74_syn_ (s, CH_3_ benzothiazine), 1.85_anti_ (s, CH_3_ benzothiazine), 4.48_anti_ (s, NH), 4.51_syn_ (s, NH), 5.29_anti_ (s, O-CH_2_), 5.31_syn_ (s, O-CH_2_), 5.60_anti_ (s, C-H benzothiazine), 5.91_syn_ (s, C-H benzothiazine), 6.71_anti_ (t, *J* = 7.5, H_7_ benzothiazine), 6.73_syn_ (t, *J* = 7.5, H_7_ benzothiazine), 6.80_anti_ (s, H_8_ chromene), 6.82_syn_ (s, H_8_ chromene), 6.87_syn_ (d, *J* = 8.5 Hz, H_6_ chromene), 6.90_anti_ (d, *J* = 8.5 Hz, H_6_ chromene), 6.93_syn_ (d, *J* = 8.0 Hz, H_3,5_ phenyl-2-oxoethoxy), 7.05_anti_ (d, *J* = 8.0 Hz, H_3,5_ phenyl-2-oxoethoxy), 7.07_anti_ (t, *J* = 7.5 Hz, H_6_ benzothiazine), 7.12_syn_ (t, *J* = 7.5 Hz, H_6_ benzothiazine), 7.33_syn_ (d, *J* = 8.5, H_5_ chromene), 7.44_anti_ (d, *J* = 8.5, H_5_ chromene), 7.52_anti_ (d, *J* = 8.5, H_3,5_ benzoyl), 7.63_syn_ (d, *J* = 8.0 Hz, H_3,5_ benzoyl), 7.64-7.67_anti_ (m, H_5,8_ benzothiazine), 7.68_syn_ (m, H_5,8_ benzothiazine), 7.72_syn_ (s, H_4_ chromene), 7.82-7.85_anti_ (m, H_2,6_ benzoyl and H_2,6_ phenyl-2-oxoethoxy), 7.85-7.89_syn_ (m, H_2,6_ benzoyl and H_2,6_ phenyl-2-oxoethoxy), 8.06_anti_ (s, H_4_ chromene); ^13^C NMR (*syn*-isomer, 125 MHz, CDCl_3_) δ 24.5, 37.6, 57.4, 70.6, 101.1, 111.8, 112.9, 116.9, 118.7, 119.3, 126.7, 128.3, 128.4, 129.5, 129.6, 129.9, 130.0, 131.9, 132.0, 132.3, 132.8, 135.5, 139.6, 140.9, 154.7, 160.6, 161.3, 191.6, 192.4; Anal. calcd for C_33_H_23_Br_2_NO_5_S: C, 56.19; H, 3.29; N, 1.99. Found: C, 56.21; H, 4.31; N, 2.09.

#### 3-(3-Methyl-2-(4-methylbenzoyl)-3,4-dihydro-2H-benzo[b][1,4]thiazin-3-yl)-7-(2-oxo-2-p-tolylethoxy)-2H-chromen-2-one (2t)

Yellow solid (397 mg, 70%); as mixture of diastereomers (*anti/syn: 30/70*); IR (KBr, cm^-1^) 3397 (NH), 1702 (C=O); ^1^H NMR (500 MHz, CDCl_3_) δ 1.74_syn_ (s, CH_3_ benzothiazine), 1.85_anti_ (s, CH_3_ benzothiazine), 2.36_anti_ (s, CH_3_ phenyl-2-oxoethoxy), 2.42_syn_ (s, CH_3_ phenyl-2-oxoethoxy), 2.44_anti_ (s, CH_3_ benzoyl), 2.45_syn_ (s, CH_3_ benzoyl), 4.50_syn_ (s, NH), 4.61_anti_ (s, NH), 5.31_anti_ (s, O-CH_2_), 5.35_syn_ (s, O-CH_2_), 5.61_anti_ (s, C-H benzothiazine), 5.96_syn_ (s, C-H benzothiazine), 6.69_anti_ (d, *J* = 2.1 Hz, H_8_ chromene), 6.72_syn_ (t, *J* = 7.3 Hz, H_7_ benzothiazine), 6.75_anti_ (t, *J* = 7.3 Hz, H_7_ benzothiazine), 6.81_syn_ (d, *J* = 2.1 Hz, H_8_ chromene), 6.87_syn_ (dd, *J* = 8.0 and 2.1 Hz, H_6_ chromene), 6.90-6.93_syn/anti_ (m, H_5,8_ benzothiazine), 7.05_anti_ (t, *J* = 7.3 Hz, H_6_ benzothiazine), 7.11_syn_ (t, *J* = 7.3 Hz, H_6_ benzothiazine), 7.17_anti_ (d, *J* = 8.0 Hz, H_3,5_ phenyl-2-oxoethoxy), 7.28-7.33 (m, H_5_ chromene (syn/anti), H_3,5_ benzoyl (syn/anti) and H_3,5_ phenyl-2-oxoethoxy (syn)), 7.34_anti_ (d, *J* = 8.0 Hz, H_2,6_ phenyl-2-oxoethoxy), 7.73_syn_ (s, H_4_ chromene), 7.79_anti_ (d, *J* = 8.0 Hz, H_2,6_ benzoyl), 7.87_syn_ (d, *J* = 8.0 Hz, H_2,6_ phenyl-2-oxoethoxy), 7.94_syn_ (d, *J* = 8.0 Hz, H_2,6_ benzoyl), 8.06_anti_ (s, H_4_ chromene); ^13^C NMR (*syn*-isomer, 125 MHz, CDCl_3_) δ 24.0, 27.7, 32.6, 43.3, 56.0, 80.2, 110.4, 110.6, 113.6, 116.1, 118.7, 118.9, 119.9, 122.4, 124.5, 126.2, 127.2, 128.4, 129.2, 131.6, 140.1, 154.2, 161.2, 161.4, 192.1, 192.6; MS, *m/z* (%) 575 (M^+^, 8%), 557 (64), 542 (43), 410 (35), 264 (44), 239 (29), 119 (100); Anal. calcd for C_35_H_29_NO_5_S: C, 73.02 ; H, 5.08; N, 2.43. Found: C, 73.21; H, 5.12; N, 2.54.

#### 3-(3-Methyl-2-(4-(methylsulfonyl)benzoyl)-3,4-dihydro-2H-benzo[b][1,4]thiazin-3-yl)-7-(2-(4-(methylsulfonyl)phenyl)-2-oxoethoxy)-2H-chromen-2-one (2u)

Yellow solid (576 mg, 82%); as mixture of diastereomers (*anti/syn: 28/72*); IR (KBr, cm^-1^) 3394 (NH), 1688 (C=O), 1320 (SO_2_), 1153 (SO_2_); ^1^H NMR (500 MHz, CDCl_3_) δ 1.78_syn_ (s, CH_3_ benzothiazine), 1.89_anti_ (s, CH_3_ benzothiazine), 3.02_anti_ (s, SO_2_-CH_3_ phenyl-2-oxoethoxy), 3.04_anti_ (s, SO_2_-CH_3_ benzoyl), 3.09_syn_ (s, SO_2_-CH_3_ phenyl-2-oxoethoxy), 3.12_syn_ (s, SO_2_-CH_3_ benzoyl), 4.50_anti_ (s, NH), 4.69_syn_ (s, NH), 5.37_syn_ (s, O-CH_2_), 5.41_anti_(s, O-CH_2_), 5.61_anti_ (s, C-H benzothiazine), 5.95_syn_ (s, C-H benzothiazine), 6.69_anti_ (s, H_8_ chromene), 6.74_syn_ (t, *J* = 7.5, H_7_ benzothiazine), 6.80_anti_ (t, *J* = 7.5, H_7_ benzothiazine), 6.83_syn_ (s, H_8_ chromene), 6.88-6.95_syn_ (m, H_6_ chromene and H_5,8_ benzothiazine), 7.08_anti_ (d, *J* = 8.0, H_6_ chromene), 7.11_anti_ (t, *J* = 7.5, H_6_ benzothiazine), 7.13_syn_ (t, *J* = 7.5 Hz, H_6_ benzothiazine), 7.36_syn_ (d, *J* = 8.0, H_5_ chromene), 7.50_anti_ (d, *J* = 8.0, H_5_ chromene), 7.59_anti_ (d, *J* = 7.5 Hz, H_8_ benzothiazine), 7.74_syn_ (s, H_4_ chromene), 7.82_anti_ (d, *J* = 7.5 Hz, H_5_ benzothiazine), 7.94_anti_ (s, H_4_ chromene), 8.04-8.19_syn/anti_ (m, H_2,3,5,6_ phenyl-2-oxoethoxy and H_2,3,5,6_ benzoyl); ^13^C NMR (*syn*-isomer, 125 MHz, CDCl_3_) δ 24.5, 44.2, 44.3, 57.5, 65.5, 70.9, 101.0, 110.9, 113.0, 116.9, 119.4, 126.9, 127.4, 127.9, 128.1, 128.5, 129.1, 129.3, 129.8, 138.0, 139.6, 140.8, 141.0, 143.9, 145.1, 154.7, 160.4, 161.2, 190.6, 192.6; Anal. calcd for C_35_H_29_NO_9_S_3_: C, 59.73 ; H, 4.15; N, 1.99. Found: C, 59.59; H, 4.31; N, 2.30.

### Pharmacology

#### Animals

Male NMRI mice weighing 20–30 g were used for studying in vivo antinociceptive activities of target compounds. Animals were maintained under standard conditions (24 ± 2°C, 60-70% humidity) and allowed food and water *ad libitum*. They were housed in appropriate cages with 12 h light/dark cycle. Before each experiment animals randomly selected and allocated into groups. The whole protocol was approved by the Ethics Committee of the Faculty of Pharmacy at Tehran University of Medical Sciences.

#### Formalin-induced pain test

All target compounds **2a-u** were subjected for testing their analgesic activity using formalin paw test [25]. The compounds or standard drug mefenamic acid were administered i.p. (30 mg/kg, 0.2 mL/20 g body weight) as a suspension in saline and tween 80 (4% w/v). Each group of mice (n = 6 animals per group) were pretreated by test compounds, mefenamic acid or vehicle, 30 minutes before injection of formalin (20 μL, 0.5%, s.c.) into the planar surface of the right hind paw. The amount of time that the animal spent licking injected paw was measured during the first 10 minutes (phase 1, neurogenic) and 10–30 minutes (phase 2, inflammatory) after formalin injection.

#### Acetic acid-induced writhing test

The analgesic activity was also determined *in vivo* by the abdominal constriction test induced by acetic acid (0.6%; 0.1 mL/10 g) in mice [21]. An acetic acid solution was administered i.p. 30 minutes after administration of compounds or mefenamic acid. After the treatment, pairs of mice were placed in separate boxes and the numbers of constrictions of the abdominal muscles, together with stretching, were counted cumulatively over a period of 60 minutes. Antinociceptive activity was expressed as the percentage of inhibition of constrictions when compared with the vehicle control group.

#### Statistical analysis

The nociception data are expressed as means ± SEM. Variance analysis (ANOVA) followed by Bonferroni’s test was used to compare means. *P*-values less than 0.05 were considered to be statistically significant.

## Results and discussion

### Chemistry

The dihydrobenzothiazole derivatives **1** were quantitatively obtained by reaction of 3-acetylcoumarins with 2-aminothiophenol derivatives in the presence of acetic acid under reflux condition or microwave irradiation [19,20]. The intramolecular Mannich-type reaction of compounds **1** with different phenacyl halides in the present of KF/Al_2_O_3_ and catalyzing by quinine hydrochloride in ethanol afforded 3,4-dihydro-2*H*-benzothiazine derivatives **2a-r** via a ring expansion. When 7-hydroxy-3-(benzothiazol-2-yl) coumarin derivative **1e** was treated with 2.5 equivalents of phenacyl halides, without protection of hydroxyl group, *O*-phenacyl derivatives **2s-u** was obtained in excellent yields (Scheme 1). The physicochemical and spectral data of new compounds **2e**, **2j-o**, and **2s-u** are described in experimental section.

### Biological activity

#### Formalin-induced nociception test

All target compounds **2a-u** were tested using formalin-induced pain test in mice [25]. The obtained results were reported as mean ± SEM of licking time and as percent of inhibition in Table 1. In general, the results showed that most of compounds were significantly able to reduce the licking time with percent of inhibition in the range of 25% to 60% at the first phase. The standard drug mefenamic acid showed 89% reduction of the licking time during the first phase. Amongst the tested compounds, **2a**, **2c**, **2f**, **2h**, **2i**, **2l-n** and **2r-u** significantly reduced the formalin induced licking time in the range of 39-98% as compared to mefenamic acid with 85% of inhibition during the second phase. Compounds **2m** and **2r-u** showed more effective antinociceptive activity in the second phase rather than first phase, indicating their ability to inhibit nociception associated with inflammatory response. Indeed, 7-hydroxy- and 7-phenacyloxy-coumarin derivatives (**2r** and **2s-u**, respectively) were more effective than mefenamic acid. Compounds **2s** and **2t** were the most effective compounds at the dose of 30 mg/kg.

**Table 1:** Antinociception activity of target compounds 2a-u and mefenamic acid (30 mg/kg, i.p.) assessed by formalin test in mice

**Compounds**	**Phase 1**			**Phase 2**		
	**Licking time**^**a**^	**Inhibition**^**b **^**(%)**	**Relative activity**^**c**^	**Licking time**	**Inhibition (%)**	**Relative activity**
**2a**	58 ± 3.46	48.10**	0.54	37.33 ± 3.93	44.28**	0.52
**2b**	51.33 ± 2.96	54.06***	0.61	50.33 ± 4.91	24.88	0.29
**2c**	68.33 ± 4.05	38.85**	0.44	38 ± 1.73	43.28**	0.51
**2d**	55 ± 2.74	50.78***	0.57	57.33 ± 6.35	14.43	0.17
**2e**	44 ± 2.89	60.63***	0.68	54 ± 4.93	19.40	0.23
**2f**	60.33 ± 3.76	46.01**	0.52	38.33 ± 4.63	42.79**	0.50
**2g**	56.66 ± 8.74	49.29**	0.55	55.66 ± 3.92	16.92	0.20
**2h**	70.25 ± 2.95	37.14**	0.42	38 ± 1	43.28**	0.51
**2i**	51.33 ± 2.40	54.06***	0.61	37 ± 1.15	44.78**	0.53
**2j**	46.25 ± 2.56	58.61***	0.66	50.33 ± 0.33	24.88	0.29
**2k**	51.66 ± 2.18	53.77***	0.60	51.66 ± 5.54	22.89	0.27
**2l**	70 ± 11.13	37.36**	0.42	37 ± 4.35	44.78**	0.53
**2m**	63.33 ± 8.21	43.33**	0.49	18.33 ± 0.33	72.64***	0.85
**2n**	69 ± 9.16	38.26**	0.43	40.66 ± 1.20	39.30**	0.46
**2o**	53.8 ± 3.21	51.85**	0.58	65 ± 6.41	2.98	0.03
**2p**	53.9 ± 3.18	51.76**	0.58	64.8 ± 4.19	3.28	0.04
**2q**	61.33 ± 5.78	45.12**	0.51	49.5 ± 2.02	26.12	0.31
**2r**	94.4 ± 4.89	25.86**	0.29	11 ± 1.7	93.64***	1.1
**2s**	50 ± 3.22	33.33**	0.37	5.2 ± 2.78	96.99***	1.14
**2t**	46 ± 2.4	38.86**	0.43	3 ± 1.04	98.26***	1.15
**2u**	34.8 ± 2.65	53.6***	0.61	14.8 ± 1.92	91.44***	1.07
Control	111.75 ± 6.94	-	-	67 ± 3.14	-	-
Mefenamic acid	12.33 ± 3.93	88.96***	1	10 ± 2.52	85.07***	1

^a^Data are expressed as mean ± S.E.M (number of animals in each group, n = 6).

^b^The percentage inhibition was determined by using the following formula: Inhibition % = 100 × (control – experiment)/control. The asterisks denote the levels of significance in comparison with control groups (*P <0.05, **P <0.01 and ***P <0.001).

^c^Activity relative to mefenamic acid was determined by using the following formula: Relative Activity = Inhibition % of compound/Inhibition % of mefenamic acid.

#### Acetic acid-induced writhing test

The analgesic activity of compounds **2b-d**, **2g-i**, **2k**, **2o** and **2r-s** was also evaluated in vivo by using abdominal constriction test induced by acetic acid in mice [21]. The abdominal constriction response induced by acetic acid is sensitive procedure to establish efficacy of peripherally acting analgesics. The analgesic activity was expressed as the percentage of inhibition of constrictions when compared with the control group. The results are summarized in Table 2.

**Table 2:** Antinociception activity of selected compounds in comparison with mefenamic acid (30 mg/kg, i.p.) assessed by acetic acid-induced writhing test in mice

**Compound**	**Nociception (Mean ± SEM)**	**Inhibition (%)**^**a**^	**Relative activity**^**b**^
**2b**	0.6 ± 0.24***	99	1.4
**2c**	38 ± 4.04***	49	0.7
**2d**	9.6 ± 2.54***	87	1.3
**2g**	3.5 ± 1.09***	96	1.37
**2h**	3 ± 1.84***	97	1.38
**2i**	4.6 ± 2***	94	1.34
**2k**	20 ± 2.48***	73	1.04
**2o**	6 ± 3.2***	92	1.31
**2r**	29 ± 2.12***	63	0.9
**2s**	14 ± 2.28***	80	1.14
**2t**	30 ± 7.6***	60	0.85
**2u**	2 ± 1.3***	98	1.4
Control^c^	75 ± 3.2		
Mefnamic acid	23 ± 1.3***	70	

^a^The percentage inhibition was determined by using the following formula: Inhibition% = 100 × (control – experiment)/control.

^b^Activity relative to mefenamic acid was determined by using the following formula: Relative Activity = Inhibition % of compound/Inhibition % of mefenamic acid.

^c^Tween 80 in saline (4% w/v).

***P <0.001 vs. control.

Significant protection against writhing was observed in animals treated with all test compounds where the mean numbers of writhes after 1 h were less than 38 compared to 75 in the control group. The percent of inhibition was in the range of 49-99%. All tested compounds were more effective than standard drug mefenamic acid with the exception of **2c**, **2r** and **2t**. Compounds **2b** and **2u** with percent of inhibition ≥98% were the most effective compounds in acetic acid-induced writhing test. Moreover, compounds **2g-i** and **2o** exhibited high protection against writhing (percent of inhibition > 90%).

#### Structure*–*activity relationships

From the structure*–*activity relationships of unsubstituted coumarin series (compound **2a-m**) based on the late stage of formalin-induced test, it was inferred that 3-thienylcarbonyl group is more favorable for activity. By comparing the activity of 7-substituted coumarin compounds **2r-u** with those of other compounds it is appeared that the 7-hydroxy or 7-phenacyloxy groups dramatically increase the effectiveness of compounds and their ability to inhibit nociception associated with inflammatory response. On contrary, compounds **2r-u** showed low level of inhibition at early phase of formalin test.

By comparing the percent of inhibition of 4-(methylsulfonyl)benzoyl derivatives **2d**, **2r** and **2u**, it is revealed that the introduction of hydroxyl group on 7-position of coumarin ring diminished the antinociception activity, while the introduction of 4-(methylsulfonyl)phenacyloxy- group increased the activity as resulted from writhing test. In the 7-phenacyloxy-coumarin derivatives **2s-u**, methylsulfonyl substituent was more favorable than bromo and methyl groups. The observed results of unsubstituted coumarin derivatives in Table 2 demonstrate that electron donating or bulky groups (for example, methoxy or phenyl, respectively) can increase antinociceptive activity in writhing test.

## Conclusion

In summary, a series of 3-(3-methyl-3,4-dihydro-2*H*-benzo[*b*][1,4]thiazin-3-yl)-2*H*-chromen-2-one derivatives **2a-u** bearing different aroyl group on the 2-position of benzothiazine ring were described as potential analgesic agents. The antinociceptive properties of target compounds were determined by formalin-induced test and acetic acid-induced writhing test in mice. The effect of substituent on aroyl moiety was explored by introduction of various electron withdrawing, electron donating or bulky groups. Surprisingly, compound **2u** bearing 2-[4-(methylsulfonyl)benzoyl]- moiety on benzothiazine ring and 4-(methylsulfonyl)phenacyloxy- group on the 7 position of coumarin nucleus showed better profile of antinoceciption in both models. It was more effective than mefenamic acid during the late phase of formalin-induced test as well as in the acetic acid-induced writhing test. However, unsubstituted coumarin derivative **2b** containing 4-methylbenzoyl moiety on benzothiazine ring, fully protected animals against writhing and was moderately able to inhibit the both phases of the formalin test. Considering the significant antinoceciptive action of phenacyloxycoumarin derivatives, compound **2u** prototype might be further used as model to obtain new more potent analgesic drugs.

## Competing interests

The authors declare that they have no competing interests.

## Authors’ contributions

MA: Synthesis of target compounds. MK: Synthesis of target compounds. SE: Collaboration in identifying of the structures of target compounds, manuscript preparation. SF: Collaboration in determination of antinociceptive properties. SFG: Collaboration in determination of antinociceptive properties. MA: Supervision of the pharmacological part, AF: Collaboration in identifying of the structures of target compounds. AS: Design of target compounds and supervision of the synthetic and pharmacological parts. All authors read and approved the final manuscript.

## Supplementary Material

Additional file 1: Table S1 Chemical structure of coumarin compounds **2a-u**. Click here for file

## References

[B1] Ruoff G Lema M Strategies in pain management: new and potential indications for COX-2 specific inhibitors J Pain Symptom Manage 2003 25 S21 S31 10.1016/S0885-3924(02)00628-0 12604154

[B2] Giovannoni MP Cesari N Graziano A Vergelli C Biancalani C Biagini P Dal Piaz V Synthesis of pyrrolo[2,3-d]pyridazinones as potent, subtype selective PDE4 inhibitors J Enzyme Inhib Med Chem 2007 22 309 318 10.1080/14756360601114700 17674813

[B3] Cesari N Biancalani C Vergelli C Dal Piaz V Graziano A Biagini P Ghelardini C Galeotti N Giovannoni MP Arylpiperazinylalkylpyridazinones and analogues as potent and orally active antinociceptive agents: synthesis and studies on mechanism of action J Med Chem 2006 49 7826 7835 10.1021/jm060743g 17181165

[B4] Magiatis P Melliou E Skaltsounis AL Mitaku S Léonce S Renard P Pierré A Atassi G Synthesis and cytotoxic activity of pyranocoumarins of the seselin and xanthyletin series J Nat Prod 1998 61 982 986 10.1021/np9800295 9722480

[B5] Beillerot A Domínguez JCR Kirsch G Bagrel D Synthesis and protective effects of coumarin derivatives against oxidative stress induced by doxorubicin Bioorg Med Chem Lett 2008 18 1102 1105 10.1016/j.bmcl.2007.12.004 18164200

[B6] Zhou X Wang XB Wang T Kong LY Design, synthesis, and acetylcholinesterase inhibitory activity of novel coumarin analogues Bioorg Med Chem 2008 16 8011 8021 10.1016/j.bmc.2008.07.068 18701305

[B7] Sashidhara KV Kumar A Kumar M Sarkar J Sinha S Synthesis and in vitro evaluation of novel coumarin–chalcone hybrids as potential anticancer agents Bioorg Med Chem Lett 2010 20 7205 7211 10.1016/j.bmcl.2010.10.116 21071221

[B8] Sashidhara KV Kumar A Kumar M Srivastava A Puri A Synthesis and antihyperlipidemic activity of novel coumarin bisindole derivatives Bioorg Med Chem Lett 2010 20 6504 6507 10.1016/j.bmcl.2010.09.055 20932744

[B9] Lee S Sivakumar K Shin WS Xie F Wang Q Synthesis and anti-angiogenesis activity of coumarin derivatives Bioorg Med Chem Lett 2006 16 4596 4599 10.1016/j.bmcl.2006.06.007 16793260

[B10] Leal LKAM Ferreira AAG Bezerra GA Matos FJA Viana GSB Antinociceptive, anti-inflammatory and bronchodilator activities of Brazilian medicinal plants containing coumarin: a comparative study J Ethnopharmacol 2000 70 151 159 10.1016/S0378-8741(99)00165-8 10771205

[B11] Keri RS Hosamani KM Shingalapur RV Hugar MH Analgesic, anti-pyretic and DNA cleavage studies of novel pyrimidine derivatives of coumarin moiety Eur J Med Chem 2010 45 2597 2605 10.1016/j.ejmech.2010.02.048 20356657

[B12] Kalkhambkar RG Kulkarni GM Kamanavalli CM Premkumar N Asdaq SMB Sun CM Synthesis and biological activities of some new fluorinated coumarins and 1-aza coumarins Eur J Med Chem 2008 43 2178 2188 10.1016/j.ejmech.2007.08.007 17959273

[B13] Ghate M Kusanur RA Kulkarni MV Synthesis and in vivo analgesic and anti-inflammatory activity of some bi heterocyclic coumarin derivatives Eur J Med Chem 2005 40 882 887 10.1016/j.ejmech.2005.03.025 16140424

[B14] Bolakatti GS Maddi VS Mamledesai SN Ronad PM Palkar MB Swamy S Synthesis and evaluation of anti-inflammatory and analgesic activities of a novel series of coumarin mannich bases Arzneim-Forsch/Drug Res 2008 58 515 520 19025062 10.1055/s-0031-1296551

[B15] Khode S Maddi V Aragade P Palkar M Ronad PK Mamledesai S Thippeswamy AHM Satyanarayana D Synthesis and pharmacological evaluation of a novel series of 5-(substituted)aryl-3-(3-coumarinyl)-1-phenyl-2-pyrazolines as novel anti-inflammatory and analgesic agents Eur J Med Chem 2009 44 1682 1688 10.1016/j.ejmech.2008.09.020 18986738

[B16] Melagraki G Afantitis A Igglessi-Markopoulou O Detsi A Koufaki M Kontogiorgis C Hadjipavlou-Litina DJ Synthesis and evaluation of the antioxidant and anti-inflammatory activity of novel coumarin-3-aminoamides and their alpha-lipoic acid adducts Eur J Med Chem 2009 44 3020 3026 10.1016/j.ejmech.2008.12.027 19232783

[B17] Rathore BS Kumar M Synthesis of 7-chloro-5-trifluoromethyl/7-fluoro/7-trifluoromethyl-4*H*-1,4-benzothiazines as antimicrobial agents Bioorg Med Chem 2006 14 5678 5682 10.1016/j.bmc.2006.04.009 16650998

[B18] Trapani G Reho A Morlacchi F Latrofa A Marchini P Venturi F Cantalamessa F Synthesis and antiinflammatory activity of various 1,4-benzothiazine derivatives Farmaco Sci 1985 40 369 376 4007156

[B19] Khoobi M Emami S Dehghan G Foroumadi A Ramazani A Shafiee A Synthesis and free radical scavenging activity of coumarin derivatives containing a 2-methylbenzothiazoline motif Arch Pharm 2011 344 588 594 10.1002/ardp.201000271 21887798

[B20] Khoobi M Ramazani A Foroumadi A Hamadi H Hojjati Z Shafiee A Efficient microwave-assisted synthesis of 3-benzothiazolo and 3-benzothiazolino coumarin derivatives catalyzed by heteropoly acids J Iran Chem Soc 2011 8 1036 1042 10.1007/BF03246560

[B21] Collier HDJ Dinnin LC Johnson CA Schneider C The abdominal constriction response and its suppression by analgesic drugs in the mouse Br J Pharmacol Chemother 1968 32 295 310 10.1111/j.1476-5381.1968.tb00973.x 4230818 PMC1570212

[B22] Tjolsen A Berge OG Hunskaar S Rosland JH Hole K The formalin test: an evaluation of the method Pain 1992 51 5 17 10.1016/0304-3959(92)90003-T 1454405

[B23] Khoobi M Ramazani A Foroumadi A Emami S Jafarpour F Mahyari A Ślepokura K Lis T Shafiee A Highly *cis*-diastereoselective synthesis of coumarin-based 2,3-disubstituted dihydrobenzothiazines by organocatalysis Helv Chim Acta 2012 95 660 671 10.1002/hlca.201100357

[B24] Victoria FN Radatz CS Sachini M Jacob RG Perin G da Silva WP Lenard EJ KF/Al_2_O_3_ and PEG-400 as a recyclable medium for the selective α-selenation of aldehydes and ketones. Preparation of potential antimicrobial agents Tetrahedron Lett 2009 50 6761 6763 10.1016/j.tetlet.2009.09.093

[B25] Hunskaar S Hole K The formalin test in mice: dissociation between inflammatory and non-inflammatory pain Pain 1987 30 103 114 10.1016/0304-3959(87)90088-1 3614974

