# Supplementary material for: Antinociceptive properties of new coumarin derivatives bearing substituted 3,4-dihydro-2H-benzothiazines
Source: Daru. 2014 Jan 7;22(1):9. doi: 10.1186/2008-2231-22-9 (PMC4029140; doi:10.1186/2008-2231-22-9)
Supplement: Additional file 1: Table S1 — Chemical structure of coumarin compounds 2a-u. [file 2008-2231-22-9-S1.docx]

**Table S1** **Chemical structure of coumarin compounds 2a-u**

| **Compound** | **R** | **Ar** |
| --- | --- | --- |
| **2a** | H | Ph |
| **2b** | H | 4-Me-Ph |
| **2c** | H | 4-Br-Ph |
| **2d** | H | 4-MeSO_2_-Ph |
| **2e** | H | 2,4-Cl_2_-Ph |
| **2f** | H | 4-NO_2_-Ph |
| **2g** | H | 4-MeO-Ph |
| **2h** | H | 4-Biphenyl |
| **2i** | H | 3-Cyclopentyloxy-4-MeO-Ph |
| **2j** | H | 4-F |
| **2k** | H | Thiophene-2-yl |
| **2l** | H | 5-Br-thiophene-2-yl |
| **2m** | H | Thiophene-3-yl |
| **2n** | 8-OMe | 4-Me-Ph |
| **2o** | 8-OMe | 4-F-Ph |
| **2p** | 8-OMe | 4-NO_2_-Ph |
| **2q** | 6-Br | 4-MeSO_2_-Ph |
| **2r** | 7-OH | 4-MeSO_2_-Ph |
| **2s** | 7-(4-Br-PhCOCH_2_O) | 4-Br-Ph |
| **2t** | 7-(4-Me-PhCOCH_2_O) | 4-Me-Ph |
| **2u** | 7-(4-MeSO_2_-PhCOCH_2_O) | 4-MeSO_2_-Ph |
